# Inhalation Exposure Study of Titanium Dioxide Nanoparticles with a Primary Particle Size of 2 to 5 nm

**DOI:** 10.1289/ehp.9469

**Published:** 2006-12-04

**Authors:** Vicki H. Grassian, Patrick T. O’Shaughnessy, Andrea Adamcakova-Dodd, John M. Pettibone, Peter S. Thorne

**Affiliations:** 1 Departments of Chemistry; 2 Chemical and Biochemical Engineering and; 3 Occupational and Environmental Health, University of Iowa, Iowa City, Iowa, USA

**Keywords:** aerosol, inhalation toxicology study, murine models, nanoparticles, nanotoxicity, particle aggregation, surface area, titanium dioxide

## Abstract

**Background:**

Nanotechnology offers great promise in many industrial applications. However, little is known about the health effects of manufactured nanoparticles, the building blocks of nanomaterials.

**Objectives:**

Titanium dioxide (TiO_2_) nanoparticles with a primary size of 2–5 nm have not been studied previously in inhalation exposure models and represent some of the smallest manufactured nanoparticles. The purpose of this study was to assess the toxicity of these nanoparticles using a murine model of lung inflammation and injury.

**Materials and Methods:**

The properties of TiO_2_ nanoparticles as well as the characteristics of aerosols of these particles were evaluated. Mice were exposed to TiO_2_ nanoparticles in a whole-body exposure chamber acutely (4 hr) or subacutely (4 hr/day for 10 days). Toxicity in exposed mice was assessed by enumeration of total and differential cells, determination of total protein, lactate dehydrogenase (LDH) activity and inflammatory cytokines in bronchoalveolar lavage (BAL) fluid. Lungs were also evaluated for histopathologic changes

**Results:**

Mice exposed acutely to 0.77 or 7.22 mg/m^3^ nanoparticles demonstrated minimal lung toxicity or inflammation. Mice exposed subacutely (8.88 mg/m^3^) and necropsied immediately and at week 1 or 2 postexposure had higher counts of total cells and alveolar macrophages in the BAL fluid compared with sentinels. However, mice recovered by week 3 postexposure. Other indicators were negative.

**Conclusions:**

Mice subacutely exposed to 2–5 nm TiO_2_ nanoparticles showed a significant but moderate inflammatory response among animals at week 0, 1, or 2 after exposure that resolved by week 3 postexposure.

Nanoscience and nanotechnology offer new opportunities for making superior materials for use in industrial and health applications (Anselmann 2001; Doumanidis 2002; Emerich and Thanos 2003; Falkenhagen 1995; Lowe 2002; McAllister et al. 2002). As these materials develop and become more widespread, there are many questions as to the consequences that nanomaterials may have on the environment. In fact it is clear from some of the recent literature that the full impact, or even partial impact, of manufactured nanomaterials on human health and the environment has yet to be fully explored (Borm 2002; Colvin 2003; Dagani 2003; Gogotsi 2003; Guzman et al. 2006; Hardman 2006; Kleiner and Hogan 2003; Masciangioli and Zhang 2003; Nel et al. 2006; Oberdörster et al. 2005a, 2005b).

Nanoparticles, the primary building blocks of many nanomaterials, may become suspended in air during production, distribution, and use. Therefore, manufactured nanoparticles can become a component of indoor and outdoor environments and thus the air we breath. Because these particles are in the respirable size range, it is important to investigate the potential health effects of these particles that are suspended in air as aerosol (Bang and Murr 2002; Oberdörster et al. 2005b; Wilson and Spengler 1996).

Commercial engineered nanoparticles join a class of particles known as ultrafine particles whose size is < 100 nm. Ultrafine particles are known to have greater adverse health effects than larger particles (Daigle et al. 2003; Oberdörster et al. 2005a; Wilson and Spengler 1996) because of their extremely high surface areas and the ability to deposit in the alveoli (Daigle et al. 2003; Oberdörster et al. 2005b; Wilson and Spengler 1996). Because manufactured nanoparticles are a specific subset of ultrafine particles, it is reasonable to surmise that they may have similar deleterious health effects if inhaled.

In this study the potential effects of manufactured nanoparticles on human health have been investigated. Here we report on an acute and subacute exposure study of titanium dioxide (TiO_2_) nanoparticles with a primary particle size between 2 and 5 nm. Although there have been earlier inhalation studies on TiO_2_ ultrafine particles (Bermudez et al. 2004), these have been conducted with particle sizes ≥20 nm. There is some evidence that TiO_2_ nanoparticles with a primary particle size < 10 nm may have chemical properties that are distinct. Studies have shown that the surface adsorption and reactivity of TiO_2_ nanoparticles approximately 6 nm in diameter were enhanced relative to nanoparticles near 16 nm in diameter. Specifically, it has been shown that the Langmuir adsorption constant, *K*_ ads_, the equilibrium constant measured for the adsorption of a series of carboxylic acids from solution on to the surface of the TiO_2_ nanoparticles was found to be much greater for the smaller TiO_2_ nanoparticles relative to the larger nanoparticles (Zhang et al. 1999). Differences in adsorption constants were for some carboxylic acids > 1,000 times for 6-nm nanoparticles, that is, *K*_ ads_ (6 nm)/*K*_ ads_ (16 nm) > 1,000. These results suggest that TiO_2_ nanoparticles < 10 nm in diameter could exhibit different properties than the nanoparticles > 10 nm that have been previously investigated in instillation and inhalation toxicologic studies.

Besides investigating the smallest commercially available TiO_2_ nanoparticles to date in an inhalation toxicology study, another unique aspect of the studies reported herein is that a number of analytical methods and techniques have been used to characterize the bulk and surface properties of the TiO_2_ nanoparticles. These analytical techniques include powder X-ray diffraction (XRD), transmission electron microscopy (TEM), Braunner, Emmett, and Teller (BET) surface area measurements, attenuated total reflection Fourier transform infrared (ATR-FTIR) spectroscopy and X-ray photoelectron spectroscopy (XPS). These well-characterized particles were used in inhalation toxicology studies. The aerosol formed in the inhalation exposure chamber was characterized further by gravimetric measurements, scanning mobility particle sizing (SMPS), and TEM so that the total mass concentration of the nanoparticles as well as the aggregation of the particles in the aerosol could be assessed. The importance of characterizing nanoparticles in health-related studies, as we have done here, has been discussed recently in detail by the International Life Sciences Institute Research Foundation/Risk Science Institute Nanomaterial Toxicity Screening Work Group (Oberdörster et al. 2005a).

## Materials and Methods

### Source of nanoparticles

We purchased the smallest commercially available TiO_2_ nanoparticles from Nanostructured and Amorphous Materials (Los Alamos, NM). The manufacturer’s specifications indicated that the powdered material is composed of TiO_2_ nanoparticles with an average primary particle size of 5 nm and a surface area of 210 ± 10 m^2^/g.

### Characterization of nanoparticles

Bulk properties were characterized by powder XRD (Bruker D-5000 q - q diffractometer with Kevex energy-sensitive detector; Bruker AXS, Inc., Madison, WI) and TEM (JEOL JEM-1230, JEOL, Ltd., Peabody, MA). Powder XRD is used to measure crystalline phase, as this technique can readily differentiate crystalline phases by the intensity of the Bragg X-ray reflections as a function of scattering angle (Atkins and de Paula 2002). Thus, for the TiO_2_ nanoparticles investigated here, the X-ray diffraction pattern measured can be compared with known diffraction patterns for the crystalline phases of TiO_2_: anatase, rutile, and brookite. TEM was used to measure primary particle size and the aggregation of the aerosol.

We used several techniques to measure surface properties, as there is some evidence that surface properties may play an important role in particle toxicity (Oberdörster et al. 2005b; Tran et al. 2000). We determined surface areas of the powders using an automated multipoint BET surface area apparatus (Nova 1200; Quantachrome Instruments, Boynton Beach, FL). Surface chemical composition and functionality were determined by XPS (custom-designed Ultra-Axis XPS system; Kratos, Manchester, UK) and ATR-FTIR spectroscopy. The ATR-FTIR measurements were made using a zinc selenide horizontal cell from Pike Technologies (Madison, WI). We placed the horizontal cell inside a Nicolet Thermo Electron FTIR spectrometer (Nexus 670; Thermo Electron Corp., Madison, WI) for these measurements.

### Exposure system—apparatus and protocol

**Figure 1:**
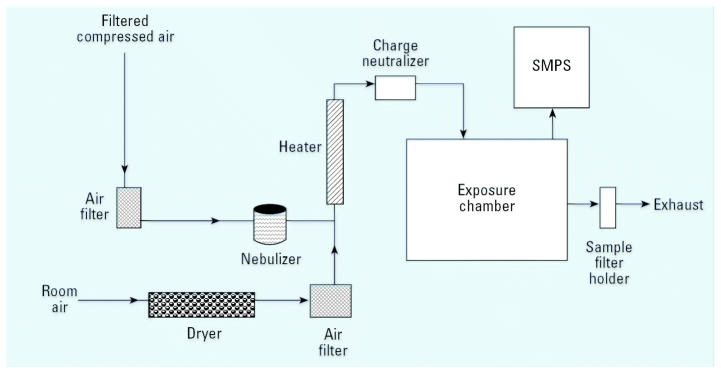
Small whole-body exposure chamber used in these studies for nanoparticle inhalation exposure studies. An aerosol-laden flow stream is generated with a nebulizer. After passing through a dryer the flow stream is sent into the exposure chamber. Nanoparticle concentrations and size distributions are measured using gravimetrical analysis and the SMPS, respectively. A TEM stub placed inside of the exposure chamber is also used for characterization. See ”Materials and Methods” for further details.

In these studies, we used a 65-L aluminum, dynamic whole body exposure chamber (O’Shaughnessy et al. 2003). This chamber was designed to operate within a standard fume hood and hold up to 24 mice in open mesh cages suspended above bedding material to maximize the free flow of particles around the mice and minimize crowding. The primary air flow rate of 25 L/min was drawn through the chamber with a rotary vane vacuum pump and measured with a calibrated rotameter (Figure 1). Inflowing air passed through a tube filled with desiccant to remove water vapor and then through a high-efficiency particulate air (HEPA) filter. We added a small blower before the desiccant tube to maintain a balanced static pressure in the chamber that was slightly positive in order to prevent room air particles from entering the chamber.

### Aerosol generation and characterization

To produce a nanoparticle aerosol, we suspended a measured amount of the bulk powder in water conditioned by reverse osmosis and ultrafiltration. Previous trials indicated that a powder concentration of 2.5 mg/mL produced an aerosol concentration of 7–10 mg/m^3^. Immediately after adding the powder to the water, the solution was sonicated by a high frequency probe (model 550; Fisher Scientific, Pittsburgh, PA) for 10 min. We then added the solution to the reservoir of a six-jet Collison nebulizer (BGI Inc., Waltham, MA). The nebulizer was operated at 20 psi from a HEPA-filtered air source. A T-connection joined the nebulizer output tube to the primary air stream. Filtered and dried air of the primary stream then carried the nebulized droplets through a heated brass pipe to completely evaporate the droplets. The dried powder aerosol then passed through a static discharge device (bipolar ion source; Simco Corp., Hatfield Township, PA) before entering the chamber.

We measured the size distribution of the aerosol in the chamber with a scanning mobility particle sizer (SMPS) consisting of a condensation particle counter (model 3010; TSI Inc., St. Paul, MN) and an electrostatic classifier with a “long” differential mobility analyzer (model 3071; TSI Inc., St. Paul, MN) that measured particles in the range of 7.5–311 nm. We calibrated the SMPS before the inhalation study with 59- and 83-nm polystyrene latex spheres. We placed copper TEM grids (400 mesh; Ted Pella, Inc., Redding, CA) in the chamber during several trial days to determine the aggregation by TEM. We measured the time-integrated mass concentration of the aerosol in the chamber by gravimetric analysis of a 47-mm glass-fiber filter placed in a stainless-steel filter holder in line with the exhaust air flow. We measured pre- and postweights with a calibrated microbalance (model MT5; Mettler-Toledo Inc., Columbus, OH) placed in a dedicated climate controlled room.

### Animals

In this study we used 6-week-old male C57Bl/6 mice (The Jackson Laboratory, Bar Harbor, ME), which were held in quarantine for 12 days before the start of exposure, in an onsite, Association for Assessment and Accreditation of Laboratory Animal Care–accredited vivarium in poly-propylene, fiber-covered cages in HEPA-filtered Thoren caging units (Thoren Caging Systems, Inc., Hazleton, PA). Mice were supplied with food (sterile Teklad 5% stock diet; Harlan, Madison, WI) and water *ad libitum* and maintained on a 12-hr light-dark cycle. The average animal weights at the time of necropsy were 22 and 25 g (in acute and subacute studies, respectively). Animal protocols were approved by the Institutional Animal Care and Use Committee and complied with the NIH *Guide for the Care and Use of Laboratory Animals* (Institute of Laboratory Animal Resources 1996).

### Inhalation exposure protocol

We exposed mice in groups of six to TiO_2_ nanoparticles for 4 hr on one occasion (acute studies) or for 4 hr/day, for 10 days (subacute studies). Mice exposed to nebulized water and sentinel mice served as controls. In the subacute study, we necropsied one group immediately after the last day of exposure (week 0), the remaining animals were euthanized in groups at weeks 1, 2, and 3 postexposure.

### Evaluation of bronchoalveolar lavage (BAL) fluid

We euthanized animals with an overdose of halothane. BAL fluid was collected, processed, and used for enumeration of total and differential cell counts as previously described (Thorne et al. 2006). The lavage supernatants were split into aliquots and frozen at −80°C for analysis of total protein, lactate dehydrogenase (LDH) activity and cytokine levels. We determined total protein using the commercially available Bradford protein assay (Bio-Rad Laboratories, Inc., Hercules, CA) with bovine serum albumin as the standard. LDH activity released from the cytosol of damaged cells into the supernatant was measured spectrophotometrically with a commercially available detection kit (Roche Diagnostics, Penzberg, Germany).

We measured the concentrations of pro-inflammatory cytokines interferon (IFN)-γ, interleukin (IL)-6, and IL-1β in the supernatants of BAL fluids using multiplexed fluorescent bead-based immunoassays (Bio-Rad Laboratories, Inc.).

### Lung histopathology

After collection of BAL fluid, we perfused lungs with 10% formaldehyde–phosphate-buffered saline (PBS) solution via the canulated trachea and stored the perfusate overnight at room temperature. The tissue was subsequently paraffin-embedded, sectioned at 5 μm, and stained with hematoxylin and eosin (H&E) and Masson’s trichrome as previously described (Thorne et al. 2006). Using routine light microscopy, a pathologist quantitatively assessed the tissue sections for histopathologic abnormalities. The histologic variables assessed included abnormalities of the parenchymal architecture (bronchioles, alveoli, pleura, vasculature); abnormal inflammatory infiltrates; presence or absence of acute lung injury; and presence or absence of fibrosis.

### Statistical analyses

We performed statistical analyses in SAS (version 9.1; SAS, Inc., Cary, NC). Values are expressed as arithmetic mean and standard error. Experimental groups were compared with control groups using the general linear model (GLM) and pairwise *t*-tests for equal or unequal variances. In all analyses, a *p*-value < 0.1 was considered suggestive of an effect and < 0.05 was considered significant.

## Results

### Particle characterization

**Figure 2:**
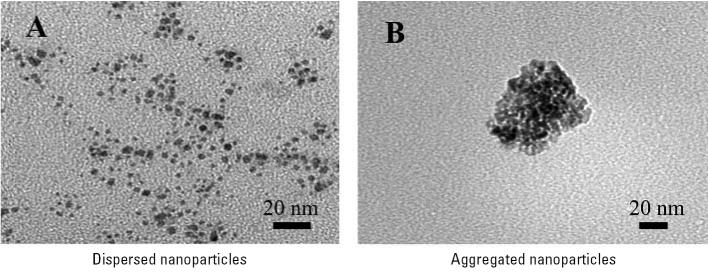
TEM images of dispersed (*A*) and aggregated (*B*) TiO_2_ nanoparticles. Dispersed nanoparticles show a primary nanoparticle size between 2 and 5 nm. For the generated aerosol, the TiO_2_ particles aggregate to form larger particles as shown in *B*.

Bulk properties such as crystallinity and particle size were characterized by powder X-ray diffraction and TEM. The X-ray diffraction pattern showed broadened lines at the expected diffraction angles for anatase with no rutile present. Anatase is the more stable form of TiO_2_ for particles < 20 nm in diameter (Naicker et al. 2005). These lines are broadened because of the small nanoparticle size. A TEM image of TiO_2_ nanoparticles after suspending the particles in methanol and sonicating for a period of time is shown in Figure 2A. The TEM image shows that the primary nanoparticle size is within a range of 2–5 nm in diameter. Analysis of 100 nanoparticles yields an average particle size of 3.5 nm with a SD of ± 1.0 nm, which is smaller than that specified by the manufacturer.

**Figure 3:**
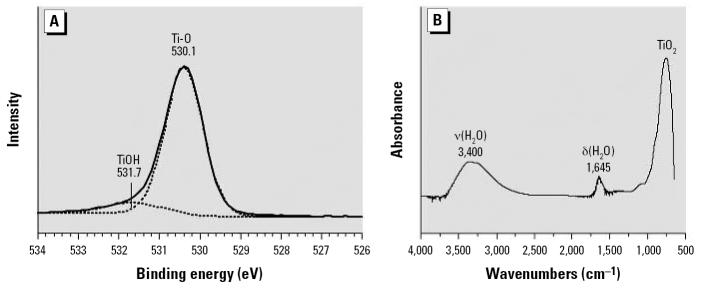
(*A*) XPS spectrum in the O(1s) region show the presence of both O atoms and O–H groups on the surface of the TiO_2_ nanoparticles. (*B*) The ATR-FTIR spectrum of TiO_2_ nanoparticles under ambient conditions. The absorption bands in the spectrum are associated with the bending, δ(H_2_O), and stretching, ν(H_2_O), modes of adsorbed water at 1,645 and 3,400 cm^−1^, respectively. The absorption band below 1,000 cm^−1^ is due to TiO_2_ lattice vibrations.

We characterized surface properties of the TiO_2_ nanoparticles. We measured the BET surface area to be 219 ± 3 m^2^/g, within the range specified by the manufacturer (210 ± 10 m^2^/g). XPS surface analysis under ultrahigh vacuum shows the presence of titanium, oxygen, and small amount of adventitious carbon in a survey spectrum (Moulder et al. 1992). A higher resolution scan in the O(1s) region (Figure 3A) showed a peak associated with surface and near-surface oxygen atoms at a binding energy of 530.1 eV. A second peak at higher binding energy, 531.7 eV, was also evident and is associated with hydroxyl, O–H groups, on the surface of the nanoparticle (Wu et al. 2005). The ATR-FTIR spectrum collected under ambient conditions is shown in Figure 3B. Three absorption bands apparent in the spectrum correspond to the bending δ(H_2_O), and stretching, ν(H_2_O), vibrations of water adsorbed on the surface of the nanoparticles at 1,645 and 3,400 cm^−1^, respectively, (Goodman et al. 2001). The absorption band below 1,000 cm^−1^ was due to oxide lattice vibrations of the TiO_2_ solid. The surface analysis data are consistent with what is known about oxide surfaces, namely they are trunicated with surface O–H groups that readily adsorb water on the surface under ambient conditions.

### In situ *aerosol characterization—SMPS and TEM data.*

We used two methods for aerosol characterization. The TEM image shown in Figure 2B is of a nanoparticle aggregate collected on the TEM stub placed in the chamber during exposure. *In situ* analysis with the SMPS revealed that the average TiO_2_ geometric mean of the mobility diameter for all measurements taken during each trial day in the subacute exposure was 128 nm with an average geometric SD of 1.7. The aerosol size distribution was consistent between days and trial types. The fact that the geometric mean diameter is much larger than the primary particle size is again proof that the TiO_2_ nanoparticles formed aggregates. An additional peak in the SMPS distribution is seen near 25 nm, which was also present for water samples that contain no TiO_2_. This peak is due to small amounts of impurities known to be present even in the purest of water (Ho et al. 1988; Krames et al. 1991).

A summary of the physicochemical data of the TiO_2_ nanoparticles and the aggregation size of the nanoparticles in the inhalation studies is given in Table 1. The exposure results and analysis are described below.

### Exposure results and analysis

#### Acute exposures

**Figure 4:**
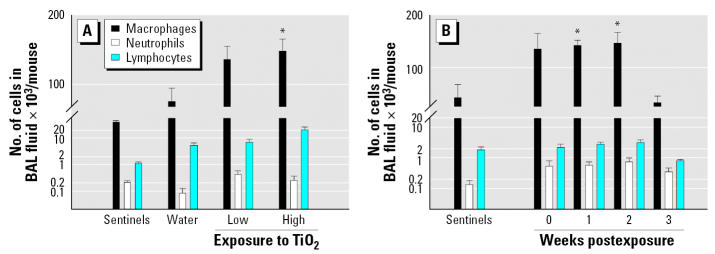
Number of macrophages, neutrophils and lymphocytes in BAL fluid among acutely (*A*) and subacutely (*B*) exposed animals. Values are expressed as mean ± SE. *Significantly different from control group, *p* < 0.05 (*t*-test for equal and unequal variances).

Mice were exposed acutely to low (0.77 mg/m^3^) or high (7.22 mg/m^3^) concentrations of inhaled TiO_2_ nanoparticles and necropsied immediately after the exposure (Table 2). The number of total cells as well as the number of macrophages was significantly (*p* < 0.05) increased in the BAL fluid of animals exposed to high concentrations of TiO_2_ particles compared with negative controls (animals exposed to aerosolized water). However, evaluation of number of neutrophils in BAL fluid (Figure 4A), total protein, activity of LDH (Table 3), and lung histopathology did not reveal evidence of inflammation.

#### Subacute exposures

**Figure 5:**
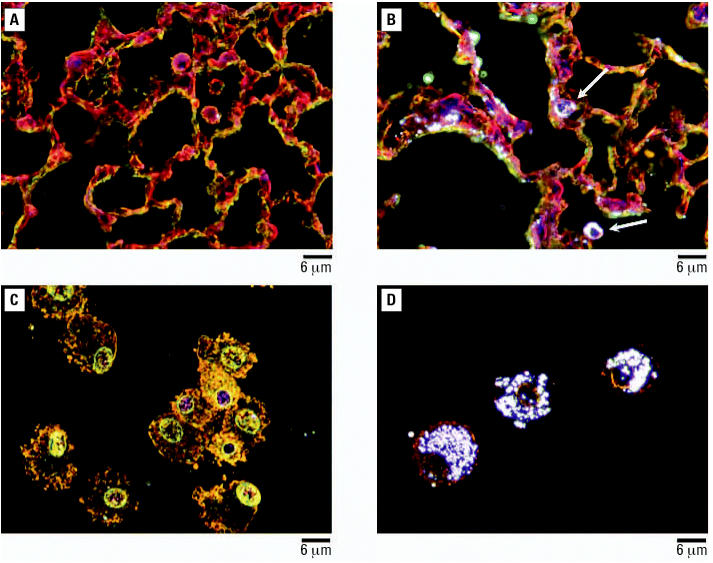
Dark field micrographs of lung tissue with H&E staining (*A,B*) and alveolar macrophages prepared by cytospinning and H&E staining (*C,D*). (*A,C*) Sentinels and (*B,D*) animals subacutely exposed to TiO_2_ nanoparticles with a primary particle size of 2–5 nm and necropsied immediately after the last exposure. Arrows point to TiO_2_ nanoparticle-laden macrophages.

**Figure 6:**
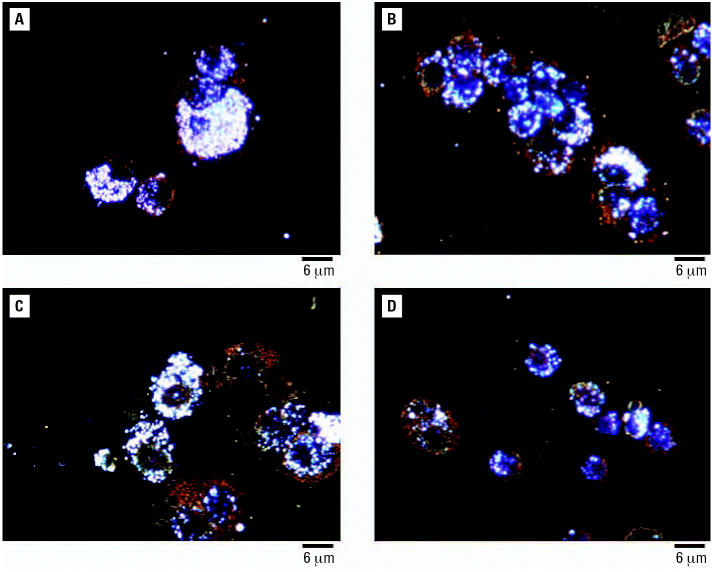
Dark field micrographs of alveolar macrophages prepared by cytospinning and H&E staining from mice exposed subacutely to TiO_2_ nanoparticles and necropsied at weeks 0 (*A*), 1 (*B*), 2 (*C*), and 3 (*D*) postexposure.

We exposed groups of mice to inhaled TiO_2_ nanoparticles 4 hr/day, for 10 days. Average concentration of nanoparticles in the whole-body exposure chamber during subacute exposures was 8.88 ± 1.98 mg/m^3^ (Table 2). Assuming a minute volume of 36 mL and deposition fraction 0.2, the cumulative inhaled TiO_2_ dose was 154 μg per mouse. All the animals exposed to TiO_2_ nanoparticles exhibited normal weight gain and behaved similarly to sentinel mice during the whole experiment. The number of alveolar macrophages was elevated in the groups of animals necropsied at weeks 0, 1, and 2 postexposure (*p* < 0.075, *p* < 0.002, and *p* < 0.018, respectively) but not in mice necropsied at week 3 postexposure (*p* < 0.753) in comparison with the sentinel group (Figure 4B). Neither neutrophils nor lymphocytes were significantly increased in the exposed groups of animals compared with sentinels. Levels of total protein and activity of LDH were not significantly different from sentinels (Table 4). Concentrations of cytokines measured in BAL fluid (IFN-γ, IL-6, and IL-1β) were very low, with most values near or below the lower limit of detection (0.14 pg/mL) and did not show significant differences among groups (Table 5). Histologic evaluation of lung tissue showed no pathologic abnormalities. Dark field microscopy revealed large alveolar macrophages with phagocytized TiO_2_ particles as shown in Figure 5. Figure 6 shows micrographs of alveolar macrophages recovered from BAL fluid at weeks 0, 1, 2, and 3 post-exposure. We have observed that macrophages were less loaded with particles at week 3 post-exposure compared with macrophages that were recovered immediately postexposure.

## Discussion

Mice exposed to TiO_2_ nanoparticles with a primary particle size of 2–5 nm showed little response to acute inhalation exposure and a modest but significant inflammatory response to subacute exposure among animals necropsied at week 0, 1, or 2 after the last exposure. Mice exposed subacutely recovered at week 3 postexposure. These manufactured nanoparticles, with the highest commercially available surface area and smallest particle size for TiO_2,_ did not show particularly toxic effects in this subacute inhalation study. In contrast, this murine model has demonstrated robust inflammatory responses upon inhalation of grain dust (Jagielo et al. 1996; Mueller-Anneling et al. 2006) or endotoxin (Thorne et al. 1999) with a high number of total cells, neutrophils, or IL-6 or TNF-α levels in BAL fluid. It should be noted, with short-term inhalation exposures in rats to iron nanomaterials, inflammatory responses were also minimal (Zhou et al. 2003).

These results are in conflict with the notion that inflammatory response is expected to be high with high surface area powders that are composed of some of the smallest nanoparticles. A surface area dependence and correlation have been observed in instillation studies (Oberdörster 2005b). However, a recent instillation study involving rats showed that the surface area for TiO_2_ nanodots and nanorods was not a significant factor in inflammatory response (Warheit et al. 2006). The nanodots had a > 6-fold increase in surface area compared with the nanorods but showed similar responses in total cell count, polymorphonuclear leukocyte percent, and BAL composition.

The resolution of inflammation after nanoparticle exposure in our inhalation studies has also been observed in other short exposure inhalation and instillation experiments of TiO_2_ (Stoeger et al. 2006). Furthermore, mice have shown quicker lung clearance than rats with TiO_2_ nanoparticles (Bermudez et al. 2004; Hext et al. 2005), thus enhancing recovery from TiO_2_ exposure.

The moderate response to exposure observed in our study may reflect a surface area threshold effect. The surface area threshold is the limit where inflammatory response occurs independently of particle size and anything below the threshold will cause little or no inflammatory response. A threshold dose has been discussed in previous studies of manganese oxide particle toxicity (Lison et al. 1997). Recently, a total surface area threshold was observed for a range of ultrafine carbonaceous particles (Stoeger et al. 2006). Stoeger et al., showed in instillation exposure studies of carbonaceous ultrafine particles there was a surface area threshold for inflammatory response for all of the different carbonaceous materials investigated. Although previous studies of TiO_2_ nanoparticles have not shown similar thresholds in instillation experiments, a total surface area threshold of 200–300 cm^2^ was found for > 2 μm particles during a chronic inhalation study (Tran et al. 2000). At present, it is difficult to compare instillation and inhalation experiments because little is known about the aggregation state of the nanoparticles in the instillation solution.

## Conclusions

In this inhalation exposure study, we have used TiO_2_ nanoparticles with a primary particle size of 2–5 nm in diameter. Detailed characterization of these nanoparticles showed a smaller size than that specified by the manufacturer, indicating that batch-to-batch variability can occur in the manufacturing of powders of oxide nanoparticles. Therefore, it seems important to perform an independent characterization of nanomaterials in toxicology studies. These nanoparticles aggregate to form an aerosol particle in the exposure chamber with a geometric mean of the mobility diameter between 120 and 130 nm. Acute exposures demonstrated no adverse effects 4 hr after the exposures commence. Analysis of lung responses in mice after subacute exposures to these aggregates showed a significant but modest inflammatory response among animals necropsied at week 0, 1, or 2 after the last exposure with recovery at week 3 post-exposure. These studies indicate that inhaled TiO_2_ nanoparticles with a primary particle size < 10 nm induce relatively modest responses and can serve as benchmark particles against which other nanomaterials can be compared.

## Figures and Tables

**Table 1:** Summary of physicochemical characterization data of TiO_2_ nanoparticles and TiO_2_ nanoparticle aerosols.

Property	Characterization
Crystalline or amorphous material	Crystalline
Phase	Anatase
Primary particle distribution	3.5 ± 1.0 nm
BET surface area	219 ± 3 m^2^/g
Surface functionalization	O, O–H, H_2_O
Aerosol size distribution, GM (GSD)	123 nm (1.6)^a^ 120 nm (1.6)^b^ 128 nm (1.7)^c^

Abbreviations: GM, geometric mean; GSD, geometric standard deviation.

a Acute exposure, low concentration.

b Acute exposure, high concentration.

c Subacute exposure.

**Table 2:** Concentration of TiO_2_ nanoparticles in whole-body chamber during exposure.

Study	Exposure group	*n*^a^
Acute	Controls	6
	0.77 mg/m^3^	6
	7.22 mg/m^3^	6
Subacute	Controls	6
	8.88 ± 1.98 mg/m^3^^b^	24

a Number of animals.

b Exposure concentrations each day: 7.78, 7.91, 7.74, 7.78, 11.16, 9.09, 7.42, 7.62, 8.78, 13.55 mg/m3.

**Table 3:** Results for the concentration of total protein and activity of LDH in BAL fluid (mean ± SE) from mice acutely exposed to TiO_2_ nanoparticles.

			TiO_2_ concentration	
	Sentinels	Water	0.77 mg/m^3^	7.22 mg/m^3^
Total protein (μg/mL)	63 ± 2	79 ± 9	91 ± 7	83 ± 3
LDH activity (U/L)	32 ± 4	36 ± 6	51 ± 16	37 ± 6

**Table 4:** Results for the concentration of total protein and activity of LDH in BAL fluid (mean ± SE) from mice subacutely exposed to TiO_2_ nanoparticles.

		Weeks postexposure			
	Sentinels	0	1	2	3
Total protein (μg/mL)	124 ± 7	112 ± 4	127 ± 16	135 ± 23	113 ± 6
LDH activity (U/L)	39 ± 9	31 ± 3	56 ± 5	57 ± 4	44 ± 10

**Table 5:** Concentration [pg/mL (mean ± SE)] of cytokines in BAL fluid from mice subacutely exposed to TiO_2_ nanoparticles.

		Weeks postexposure			
Cytokine^a^	Sentinels	0	1	2	3
INF-γ	0.15 ± 0.01	0.22 ± 0.06	0.23 ± 0.06	0.14 ± 0.00	0.15 ± 0.01
< L LOD/*n*	5/6	3/6	2/6	6/6	5/6
IL-6	0.50 ± 0.22	0.84 ± 0.49	0.22 ± 0.04	0.43 ± 0.09	0.27 ± 0.04
< L LOD/*n*	3/6	2/6	3/6	0/6	1/6
IL-1β	1.03 ± 0.27	0.63 ± 0.04	1.03 ± 0.47	0.51 ± 0.11	0.52 ± 0.18
< L LOD/*n*	0/5^b^	0/4^b^	0/6a	0/5^b^	2/6

a Lower limit of detection (L LOD) is 0.14 pg/mL for all three cytokines.

b Four mice with hemorrhagic BAL fluid were excluded (one animal from the sentinel group, two from week 0 postexposure, and one from the group necropsied at week 2 postexposure).
